# How Voluntary Workers and the State Can Help

**Published:** 1920-07-31

**Authors:** 


### THE DEVON JOINT COMMITTEE.

### How Voluntary Workers and the State can Help.

The first meeting of the Devon Joint Committee under the Council of the Red Cross and the Order of St. John for the co-ordination of hospital and nursing services was lately addressed by Sir Napier Burnett, Director of Medical Services under the Ministry of Health.

Lord Fortescue presided and stated that the new committee formed to make the voluntary aid services permanent consisted of 45 members, most of whom were former officials of the Red Cross, together with representatives of the county and borough councils of Exeter, Plymouth and Torquay, the hospitals, nursing associations and medical officers of health.

Sir Napier Burnett analysed the hospital situation in the country. The proportion of beds in the rural districts is fairly good, but in the towns it fell short of the desirable ratio of three per thousand. The Poor-Law infirmaries in the county are more than half empty. In one city a thousand beds are empty, while a civil hospital there has a long waiting-list. The Minister of Health should adjust these differences. He has made a survey of the finances of the forty hospitals of the county of which thirty-six gave returns one reported no deficit; one was closed; the remaining thirty-four, up to the end of 1918, are financially healthy. In 1919 the prevailing stringency appeared. In twelve hospitals working-class patients paid nearly £4,000, and he thought that this source of income could be developed.

The alternative to nationalisation or municipalisation is the voluntary system with State assistance in the form of capital expenditure. He preferred this to subsidies because a State subsidy at 3s. or 4s. per occupied bed would tend to rise to 5s. or 6s. in the next two years, when very soon voluntary subscriptions would diminish, and national hospitals would be forced upon them.

The new committee can do useful work in helping to organise regular collections from employers and workpeople, which he much prefers to spasmodic appeals with the sploshy sentiment involved in most of them. How futile they sometimes are can be shown in the case of an appeal sent from a Plymouth hospital, which was sent to 26,000 persons and resulted in £500 from donations and £50 in annual subscriptions! Next to the patients, the insurance companies gain most from the work of hospitals, and they have not yet acknowledged its value. A penny a week from every adult, a halfpenny from every child in each occupied house in the county would raise £50,000, and meet all the requirements of the Devonshire hospitals.

